# Spatiotemporal Gait Parameters as Predictors of Lower-Limb Overuse Injuries in Military Training

**DOI:** 10.1155/2016/5939164

**Published:** 2016-07-13

**Authors:** Shmuel Springer, Uri Gottlieb, Mariya Lozin

**Affiliations:** ^1^Department of Physical Therapy, Faculty of Health Science, Ariel University, Ariel, Israel; ^2^Israel Defense Force Medical Corps, Zerifin, Israel

## Abstract

The study objective was to determine whether spatiotemporal gait parameters could predict lower-limb overuse injuries in cohort of combat soldiers during first year of military service. Newly recruited infantry soldiers walked on a treadmill at a 15° incline with a fixed speed of 1.67 m/sec while wearing a standard military vest with a 10 kg load. Stride time variability, stride length variability, step length asymmetry, and the duration of the loading response phase of the gait cycle were measured. Injury data on 76 soldiers who did not report musculoskeletal complaints at initial screening were collected one year after recruitment. Multiple logistic regression analyses were conducted to determine the predictive effect of the gait parameters on lower-limb injuries. Twenty-four soldiers (31.6%) had overuse injuries during the first year after recruitment. Duration of the loading response was a significant predictor of general lower-limb injury (*p* < 0.05), as well as of foot/ankle and knee injuries (*p* < 0.05, *p* < 0.01, resp.). A cutoff value of less than 12.15% for loading response duration predicted knee injuries with 83% sensitivity and 67% specificity. This study demonstrates the utility of spatiotemporal gait evaluation, a simple screening tool before military training, which may help to identify individuals at risk of lower-limb overuse injuries.

## 1. Introduction

Musculoskeletal overuse injuries are most common among initial military recruits. These injuries, which have been termed a hidden epidemic, are extremely costly in terms of training time and military expenses and have impact on military readiness [1–5]. Specifically, the lower-limb is at particular risk of overuse injuries, most of which occur at or below the knee, with the most common injuries include patellofemoral pain, medial tibial stress syndrome, Achilles tendinopathy, and plantar fasciitis/plantar heel pain [6–8].

Marching and walking for long distances are key components of basic military practice and are highly related to musculoskeletal overuse injuries [9, 10]. The highest incidence of injuries during basic military training is typically recorded during training characterized by marching and prolonged walking [10]. In addition, walking with an added load is an inevitable part of the daily schedule of a soldier and an important part of training. Vigorous military load marching and walking over uneven or inclined terrain are commonly implicated in these injuries [11–13].

Alterations in movement pattern may expose military personnel to increased risk for overuse injuries. Wang et al. [14] reported significant changes in lower-extremity joint mechanics, such as increased pelvic tilt, increased knee and hip flexion, and decreased ankle dorsiflexion at heel strike, during loaded and fatigued walking. In addition, it was demonstrated that load carriage has a significant influence on peak ground reaction forces and loading rates [15]. It should be noted that a systematic review by Zadpoor and Nikooyan [16] indicated that loading rate was found to be significantly different between stress fracture and control groups, with the loading rate significantly higher in the stress fracture group.

Recent evidence suggests that analysis of movement patterns may not only reflect deviations resulting from complex walking tasks, but also be used to predict musculoskeletal injuries [17–19]. Thijs et al. [18] reported that measurements of plantar pressure distribution during initial contact and loading response may predict anterior knee pain. Willems et al. [19] prospectively determined gait-related risk factors for exercise-related lower leg pain (ERLLP) in 400 students. Their analysis revealed that subjects who developed ERLLP had an altered running pattern before the injury compared to controls. In addition, there is a growing body of evidence suggesting that variability or fluctuation of gait is protective for overuse injuries [20]. Mann et al. [21] compared male and female runners who did or did not sustain an overuse injury during a one-year period. The previously uninjured runners displayed significantly greater stride to stride variability. Similarly, participants with chronic ankle sprains demonstrated less stride to stride variability compared to healthy controls [22].

Given the alternate gait mechanism demonstrated during loaded and strenuous walking and the ability to utilize gait parameters as predictors of musculoskeletal injuries, it was speculated that examining the gait pattern during this task may help to identify soldiers at risk for lower-limb injury. Therefore, the aim of this study was to investigate the predictive value of spatiotemporal gait parameters measured during loaded and strenuous walking, for lower-limb overuse injuries in combat soldiers during the first year of military service.

## 2. Methods

As part of the Israel Defense Forces (IDF) Medical Corps agenda to implement preventive medicine strategies to reduce the rate of overuse injuries new infantry recruits (up to six weeks from recruitment) go through a screening evaluation at IDF Warrior Health Center (WHC). The screening is intended to identify individuals needing special training or exercise programs and it comprised a questionnaire designed to obtain information about musculoskeletal complaints or injuries, an assessment by a physical therapist, and gait test. The gait test is performed while the subject walks on a treadmill at a 15° incline, with a fixed speed of 1.67 m/sec (i.e., 6 km/h) for 5 minutes, while carrying a standard military vest with a total load of 10 kg. It should be noted that a fixed speed of 1.67 m/sec (6 km/h) was chosen as it represents a typical speed of military marching. The speed was not adjusted to height since in military setting soldiers are required to march in arbitrary conditions with a fixed speed, regardless of their anthropometrics. In addition, such protocol attributes to overall increase in work requirements from various muscles and challenges overall balance [23].

During the gait test spatiotemporal parameters are measured using the OptoGait system (Microgate, Bolzano, Italy); data is sampled at 1000 Hz and processed using dedicated software (Optojump Next, Version 1.3.20.0, Microgate, Bolzano, Italy). The OptoGait system consists of transmitter and receiver bars, one-meter each, located on both sides of the treadmill. The transmitter bar has 99 infrared LEDs while the receiver bar has 99 sensors. Stepping in between the bars blocks the infrared rays, allowing the system to obtain spatiotemporal gait parameters without the use of additional markers. The OptoGait system demonstrated high validity and reliability for the assessment of gait parameters compared with a validated electronic walkway (GAITRite) in young and older subjects as well as in orthopedic patients [24, 25].

The present report is a retrospective cohort investigation of six platoons of infantry soldiers recruited to the IDF that were screened at the WHC. The study was approved by the Israel Defense Force Medical Corps Ethical Review Board (approval number IDF-1497-2015).

One year after recruitment, data regarding lower-limb overuse injuries were collected from the soldiers' electronic medical records. Injury was defined as at least two complaints in the same area of the lower-limb with a diagnosis provided by the military base medical physician or one complaint that resulted in a physician's recommendation to abstain from at least two days of training [26]. The injury data was extracted independently by two physical therapists (Shmuel Springer and Uri Gottlieb) that are familiar with overuse injuries. Any disagreements were checked against the original medical record. The injuries were classified according to three key sites: knee overuse injuries (e.g., patellar tendonitis, iliotibial band syndrome); tibial overuse injuries (e.g., stress fractures, medial tibial stress syndrome); and foot/ankle overuse injuries (e.g., Achilles tendonitis, plantar fasciitis). Participants may have experienced more than one injury but were only counted once in the injured group. In addition, soldiers that reported musculoskeletal complaints or injuries in their lower-limb or back at initial screening were excluded from the study.

### 2.1. Statistical Analysis

Four spatiotemporal gait parameters were defined: stride time variability [100 × (standard deviation of stride time/mean stride time)], stride length variability [100 × (standard deviation of stride length/mean stride length)], step length asymmetry [100 ×  {ln × (step length right/step length left)}], and the duration of the loading response phase of the gait cycle (initial contact of the ipsilateral foot to the swing phase by the contralateral foot). Descriptive statistics were used to summarize subject demographics, lower-limb injuries, and means and standard deviations (SD) of the gait parameters. Examination of statistical assumptions demonstrated strong correlation between the durations of the loading responses at the right and left limbs. Thus, to avoid multicolinearity, the left loading response parameter was excluded from analyses.

A logistic regression analysis using the Wald test and backward elimination method was conducted to determine the predictive effect of the gait parameters on general lower-limb injury occurrences and at each of the three key sites (i.e., knee, tibia, and foot/ankle). Stepwise linear regression was carried out to predict the total injuries per subject. In addition, ROC (Receiver Operating Characteristic) analysis was used to examine the AUC (area under the curve), sensitivity, specificity, and cutoff (based on Youden index) [27] of the strongest predictors. Significance was set at *p* < 0.05. IBM SPSS 22.0 software was used for statistical analyses.

## 3. Results

A total of 76 IDF male soldiers were included in the analysis. Of these, 24 (31.6%) had lower-limb overuse injuries during the first year after recruitment, and 52 (68.4%) were uninjured. Of the injured group, 12 (15.8%) had a foot/ankle injury, 5 (6.6%) had a tibial injury, and 12 (15.8%) had a knee injury. Characteristics of the cohort are summarized in Table 1. No significant differences were found between the injured and uninjured groups in demographic measures of age, height, weight, and BMI.

Table 2 presents descriptive statistics of gait parameters. The results of all stepwise binary logistic regression analyses are found in Table 3. The stepwise binary logistic regression analyses performed to determine the relative predictive value of the four gait parameters for general lower-limb overuse injury occurrence yielded only two significant parameters (*χ* ^2^ = 9.06, *df* = 1,  *R* ^2^ = 15.8%, *p* < 0.05), the duration of the loading response (*p* < 0.05) and stride time variability (*p* < 0.05, one-tailed). The three separate stepwise binary logistic regression models performed to determine the relative predictive value of the gait parameters for injury occurrence at each of the three key sites (i.e., knee, tibia, and foot/ankle) indicated significant results for the duration of the loading response as a predictor for foot/ankle injuries (*χ* ^2^ = 5.54, *df* = 1, *R* ^2^ = 12.1%, *p* < 0.05) and for knee injuries (*χ* ^2^ = 7.29, *df* = 1, *R* ^2^ = 15.7%, *p* < 0.01). The logistic regression for tibial injuries did not reveal any significant predictor.

The stepwise linear regression analysis with the gait parameters as predictors and the sum of total injuries as a dependent variable indicated that the duration of the loading response was the only significant predictor (*F*(1,74) = 7.36, *R* ^2^ = 9%, *β* = −0.3, *p* < 0.01).

A univariate ROC analysis was used to determine an optimal cutoff score of the duration of the loading response for injured and uninjured soldiers in the logistic regression models which revealed it as a significant predictor. For general lower-limb injury, ROC analysis yielded an AUC of 0.65, sensitivity (*S*_*n*_) of 63%, and specificity (*S*_*p*_) of 69%, with an optimal cutoff value of 12.15%. For knee injuries, ROC analysis yielded an AUC = 0.75, *S*_*n*_ = 83%, and *S*_*p*_ = 67% with the same optimal cutoff value of 12.15%. For foot/ankle injuries, ROC analysis yielded an AUC of 0.67, *S*_*n*_ = 67%, and *S*_*p*_ = 78% with optimal cutoff value of 11.35%. It is important to note that the cutoff value of 12.15% matched the values of prior ROC analyses and had same sensitivity of 67%, but lower specificity of 64%.

## 4. Discussion

Based on the understanding that injuries were becoming a major cause of morbidity in the military services, the US Armed Forces Epidemiological Board (AFEB) formed an expert work group intended to provide recommendations for injury prevention. The work group indicated that research should be conducted to identify risk factors for injuries [4]. Our results suggest that spatiotemporal gait evaluation may assist in identifying soldiers at higher risk of developing lower-limb overuse injuries. Specifically, the current findings indicated that loading response duration of less than 12.15% of the gait cycle, during loaded and strenuous walking on an inclined surface, may predict knee injuries with 83% sensitivity and 67% specificity and foot/ankle injuries with 67% sensitivity and 64% specificity. To the best of our knowledge, this is the first study that demonstrates that spatiotemporal gait parameters can predict overuse injuries among soldiers. Loading response, which at a usual rate of walking is 12% of the gait cycle, represents one of the most demanding tasks during gait, requiring a great deal of coordination, shock absorption, and limb stability [28].

Several factors should be considered in interpreting the data from this study. The short loading response may be associated with weak or fatigued dorsiflexor muscles, which cannot absorb shock and dissipate ground reaction forces. Thus, ground reaction forces are transmitted to the bone, increasing the risk of injury [16, 29]. Furthermore, fatigued dorsiflexors may lead to decreased knee flexion, consequently decreasing the attenuation of the impact acceleration caused by foot strike [30]. Another possible explanation for shorter loading response values might be limited dorsiflexion range of motion in the contralateral leg during preswing, making the ipsilateral leg pass faster until reaching the floor. This assumption is supported by previous research showing associations between limited ankle joint dorsiflexion range of motion (ROM) and overuse injuries [31, 32]. It should be also noted that ambulation on an inclined surface, as in the present study, requires increased ankle dorsiflexion and greater muscle activation of the knee and ankle joints for stability [33].

Our results appear to support previous investigations that demonstrated an association between lower-limb injuries and altered loading response [16, 29]. However, while previous studies used myoelectric signals or ground reaction force kinetics, the current report demonstrates that even a simple, low cost, and easy to administer spatiotemporal gait evaluation that mimics typical military marching (i.e., slope, load carriage) may be a beneficial screening tool to identify soldiers at risk for overuse injuries. Nevertheless, it is unclear whether these risk factors can be identified clinically without any equipment. The current study should encourage further investigations in this direction.

One possibility for a simple screening tool might be exploring the correlation between the loading response phase and other factors, such as dorsiflexor strength and ROM. In addition, clinicians and trainers may consider incorporating programs that enhance the motor control over the ankle (i.e., ROM, dorsiflexor strength, and endurance) aiming to reduce the risk for injury. For example, it has been shown that heel lifts increase ankle dorsiflexion excursion and time to heel off during ambulation in subjects with limited dorsiflexion ROM [34].

While the duration of the loading response was a significant predictor of injury, among the other three parameters (e.g., stride time variability, stride length variability, and step length asymmetry), only stride time variability demonstrated an association with injury risk. It has been suggested that variability may assist with flexible adaptation to stresses placed on the human body [35]. Additional research is needed to determine the role of gait variability as a predictor of overuse injuries. In addition, the inconclusive results regarding step length asymmetry in the present study are consistent with recent data suggesting that functional difference does not appear to be the consequence of abnormality but rather relates to the contribution of each limb to propulsion and control tasks [36].

Several factors should be considered in interpreting the data from this study. The study design was retrospective and the cohort consisted of a relatively small sample that included only males. Future prospective studies with more participants, including female soldiers, are warranted. Another limitation is that the gait evaluation was conducted for a short period and the load carried by the soldiers was relatively light. A more strenuous protocol with prolonged walking and heavier load, causing more gait alterations, may be required to fully explore the role of spatiotemporal parameters as predictors of injuries. Finally, previous injuries are major risk factors of overuse injuries. Thus, soldiers that reported musculoskeletal complaints or injuries in their lower-limb or back at initial screening were excluded from the study cohort. Nevertheless, there are other known risk factors of overuse injuries such as a pronated foot type, low level of previous physical activity, and smoking [1], which were not controlled in the present study. Future investigations should confirm the present results while controlling for other known risk factors.

## 5. Conclusions

The current study has demonstrated that spatiotemporal gait evaluation during loaded and strenuous walking on an inclined surface may be a simple screening tool to facilitate identification of soldiers at risk of overuse injuries. A <12.15% duration of the loading response phase of the gait cycle was associated with knee and foot/ankle injuries.

Future research should evaluate this screening method with larger, more varied, prospective military cohort.

## Figures and Tables

**Table 1:** Characteristics of the included cohort means and standard deviations (M ± SD).

Characteristic	Total (*N* = 76)	Injured (*n* = 24)	Uninjured (*n* = 52)	*p* value
Age (yrs)	19.53 ± 1.29	19.72 ± 1.36	19.45 ± 1.27	0.42
Height (cm)	174.82 ± 5.18	175.63 ± 5.2	174.44 ± 5.18	0.19
Weight (kg)	71.87 ± 10.88	72.77 ± 9.14	71.46 ± 11.66	0.48
BMI	23.48 ± 3.11	23.56 ± 2.52	23.44 ± 3.36	0.84
Total injuries	0.38 ± 0.61	1.21 ± 0.41	—	—

**Table 2:** Gait parameters means and standard deviations (M ± SD).

Gait parameter	Total (*N* = 76)	Injured (*n* = 24)	Uninjured (*n* = 52)	*p* value
Stride time variability (%)	2.5 ± 0.86	2.17 ± 0.51	2.63 ± 0.96	0.04
Stride length variability (%)	2.76 ± 1.08	2.61 ± 0.95	2.82 ± 1.14	0.43
Step length asymmetry index	3.23 ± 2.54	2.77 ± 2.09	3.45 ± 2.72	0.35
Load response Rt (%)	12.14 ± 3.79	10.68 ± 4.34	12.82 ± 3.33	0.02

**Table 3:** Summary of binary logistic regression analyses with backward elimination method.

	General lower-limb injury occurrences	Ankle & Foot injury	Knee injury	Tibia injury^a^
Loading response (OR)	0.87^*∗*^	0.83^*∗*^	0.81^*∗∗*^	—
Stride time variability (OR)	0.45^b^	—	—	—
Stride length variability (OR)	—	—	—	—
Step length asymmetry index (OR)	—	—	—	—
Concordance (%)	73.7	84.2	84.2	—
Nagelkerke *R* ^2^ (%)	15.9^*∗*^	12.1^*∗*^	15.7^*∗∗*^	—

OR: odds ratio; ^a^all variables were removed from the regression model; ^*∗*^ *p* < 0.05; ^*∗∗*^ *p* < 0.01; ^b^ *p* < 0.05 (one-tailed).
